# A Mobile Peer Intervention for Preventing Mental Health and Substance Use Problems in Adolescents: Protocol for a Randomized Controlled Trial (The Mind Your Mate Study)

**DOI:** 10.2196/26796

**Published:** 2021-07-30

**Authors:** Louise Birrell, Ainsley Furneaux-Bate, Cath Chapman, Nicola C Newton

**Affiliations:** 1 The Matilda Centre for Research in Mental Health and Substance Use The University of Sydney Sydney Australia

**Keywords:** prevention, mental health, substance use, peer support, depression, anxiety, help-seeking, mobile phone

## Abstract

**Background:**

Anxiety, mood, and substance use disorders have significant social and economic impacts, which are largely attributable to their early age of onset and chronic disabling course. Therefore, it is critical to intervene early to prevent chronic and debilitating trajectories.

**Objective:**

This paper describes the study protocol of a CONSORT (Consolidated Standards of Reporting Trials)-compliant randomized controlled trial for evaluating the effectiveness of the Mind your Mate program, a mobile health (mHealth) peer intervention that aims to prevent mental health (focusing on anxiety and depression) and substance use problems in adolescents.

**Methods:**

Participants will consist of approximately 840 year 9 or year 10 students (60 students per grade per school) from 14 New South Wales high schools in Sydney, Australia. Schools will be recruited from a random selection of independent and public schools across the New South Wales Greater Sydney Area by using publicly available contact details. The intervention will consist of 1 introductory classroom lesson and a downloadable mobile app that will be available for use for 12 months. Schools will be randomly allocated to receive either the mHealth peer intervention or a waitlist control (health education as usual). All students will be given web-based self-assessments at baseline and at 6- and 12-month follow-ups. The primary outcomes of the trial will be the self-reported use of alcohol and drugs, anxiety and depression symptoms, knowledge about mental health and substance use, motives for not drinking, and willingness to seek help. Secondary outcomes will include positive well-being, the quality of life, and the impact of the COVID-19 pandemic. Analyses will be conducted using mixed-effects linear regression analyses for normally distributed data and mixed-effects logistic regression analyses for categorical data.

**Results:**

The Mind your Mate study was funded by an Australian Rotary Health Bruce Edwards Postdoctoral Research Fellowship from 2019 to 2022. Some of the development costs for the Mind your Mate intervention came from a seed funding grant from the Brain and Mind Centre of the University of Sydney. The enrollment of schools began in July 2020; 12 of 14 schools were enrolled at the time of submission. Baseline assessments are currently underway, and the first results are expected to be submitted for publication in 2022.

**Conclusions:**

The Mind your Mate study will generate vital new knowledge about the effectiveness of a peer support prevention strategy in real-world settings for the most common mental disorders in youth. If effective, this intervention will constitute a scalable, low-cost prevention strategy that has significant potential to reduce the impact of mental and substance use disorders.

**Trial Registration:**

Australian New Zealand Clinical Trials Registry ACTRN12620000753954; https://www.anzctr.org.au/Trial/Registration/TrialReview.aspx?id=379738&isReview=true

**International Registered Report Identifier (IRRID):**

DERR1-10.2196/26796

## Introduction

### Background

By 2030, the global health care costs associated with mental illness and substance use are predicted to be more than the pooled costs attributed to cancer, diabetes, and respiratory diseases [1]. Although the provision of widespread treatment is essential, if we are to significantly affect population health, effective prevention is also critical. Recent population data show that mental health problems among adolescents are rising [2], indicating that current prevention approaches do not prevent population level increases. Both mental health symptoms and substance use, begin and escalate during adolescence [3], with even small elevations in adolescence increasing the likelihood of developing a full-blown mental disorder later in life [4]. From the ages of 13 to 24 years, there is an increased susceptibility to the development of mental illness and substance use problems, with longitudinal life course studies indicating that transitions from childhood to adolescence and from adolescence to young adulthood are marked by significant increases in mental and substance use disorders [3]. This makes adolescence a critical time to intervene and prevent these problems using scalable and novel prevention approaches.

The social network theory has demonstrated that peers have a powerful influence on health behaviors, including mental health [5] and substance use [6]. Studies using social network analysis are beginning to uncover the mechanisms by which health behaviors, such as mental health symptoms and alcohol use, diffuse and spread through peer groups [7-9]. This approach is currently used to better understand the development and prevention of these problems. Several studies using a large nationally representative US data set (AddHealth) found that both friendship connections and peer influence function are causal factors that influence adolescents’ health risk behaviors [10,11]. The size of this influence is substantial, with a 10% increase in the proportion of adolescent peer group members who drink alcohol, resulting in a 4% increase in individual drinking [12]. Furthermore, social connections among peers are important determinants of behavior, with a one SD increase in (1) friends’ prior drinking increasing the odds of an adolescent binge drinking by 30% and (2) friends-of-partner drinking increasing the odds of binge drinking by 81% [13]. Research has also started to uncover the role of peer groups in the etiology of mental health symptoms, with processes such as corumination between peers actively increasing the risk of developing poor mental health, such as depression [14,15]. Conversely, it has recently been speculated that interventions providing positive support and scaffolding through the power of peers to develop prosocial, healthy behaviors during adolescence could have significant benefits for adolescent health and development [16].

Current prevention programs for adolescent mental health and substance use typically show modest effect sizes and limited implementation [17]. Universal school-based programs delivered to all adolescents, regardless of the level of risk, have been shown to demonstrate small to moderate effects in reducing alcohol misuse [18,19] and levels of anxiety and depression [20,21] and increasing mental health literacy [22]. As many current mental health prevention approaches largely represent adaptations of treatment approaches that were originally designed for adults, it is unclear how the unique developmental needs of adolescents have been taken into account in the design of these programs [23]. Adolescents are hypersensitive to social stimuli and are more susceptible to peer feedback on decision-making. Therefore, it is critical to consider the substantial role of peers in designing effective prevention strategies [23]. Furthermore, a recent review [17] of mental health interventions in schools in *The Lancet* identified the development and evaluation of peer-led models as a significant gap in the field.

In addition to influencing behavior, peers play an important role in identifying and facilitating access to support for friends with mental health and substance use issues. Currently, most young people experiencing difficulties with mental health and/or substance use will not seek help [24]. One study found that approximately only 20% of adolescents (aged 14-15 years) sought help for depression, whereas only 3% sought help for alcohol or other drug problems [25]. Those who sought help for substance use cited that friends were their main source of support, followed by parents or health professionals as reported by a much smaller proportion [25]. However, adolescents may not be equipped with the knowledge, skills, or awareness of appropriate referral services to provide adequate support to their peers when mental health and substance use problems arise. Thus, empowering adolescents to help their peers is not only critical to ensure that the advice and support received is appropriate, but can also facilitate help seeking for those providing support [25].

One existing, evaluated peer training program for adolescents is Teen Mental Health First Aid. Developed in Australia, this program includes three in-school sessions with trained facilitators to increase mental health literacy and reduce stigma, with separate courses for students in years 7-9 (ages 12-14) and years 10-12 (ages 15-17) [26,27]. These programs have shown promising effects in increasing help-seeking intentions and confidence to support friends and reducing stigma around mental health [26,27]. However, the program’s impact on mental health symptoms has yet to be evaluated and it uses face-to-face delivery from external trainers, potentially limiting its scalability.

Current adolescents have grown up in a world with unequivocal access to technology and information. In Australia, 91% of teenagers (aged 14-17 years) own a mobile phone, and 94% of these phones are internet-connected smartphones [28]. One key opportunity for the prevention of mental disorders is the possibility of harnessing such mobile technology to deliver easily accessible prevention strategies that can be widely implemented at low cost, in a format that is acceptable to and engaging for adolescents. Adolescents use their phones to communicate with their peers daily and regularly access web-based information. In a US survey, 89% of teenagers reported being on the web *several times a day* to *constantly* [29], with SMS text messaging as the dominant form of communication among peers on a daily basis [30]. Despite the recognition of the key role that peers play in health behavior and the frequent use of digital technologies by adolescents, scientifically evaluated digital peer interventions are lacking, with limited evaluations of current web-based or mobile programs available for adolescents.

To address these gaps, the Mind your Mate intervention was developed, a novel mobile health (mHealth) intervention aimed at upskilling and empowering adolescents to better support their peers on issues related to mental health, alcohol, and other drug use.

### Aims, Objectives, and Hypotheses

This study aims to evaluate the effectiveness of Mind your Mate program among adolescents through a cluster randomized controlled trial (RCT) conducted in partnership with secondary schools. We hypothesized that the Mind your Mate intervention will be more effective than the active control group (health education as usual) in (1) delaying the uptake of alcohol and other drugs; (2) reducing anxiety and depression symptoms; (3) increasing knowledge about mental health, alcohol and other drugs; and (4) increasing help-seeking behavior.

## Methods

### Ethics Approval and Consent to Participate

The study was approved by the University of Sydney Human Research Ethics Committee, Australia (project number: 2020/054). Both active parental consent and active participant (student) consent were required before students could participate in the study.

Access to schools was granted by the New South Wales (NSW) Department of Education and Communities (State Education Research Applications Process 2020130).

### Study Design

This trial is registered with the Australian New Zealand Clinical Trials Registry (trial number: ACTRN12620000753954).

**Figure 1:**
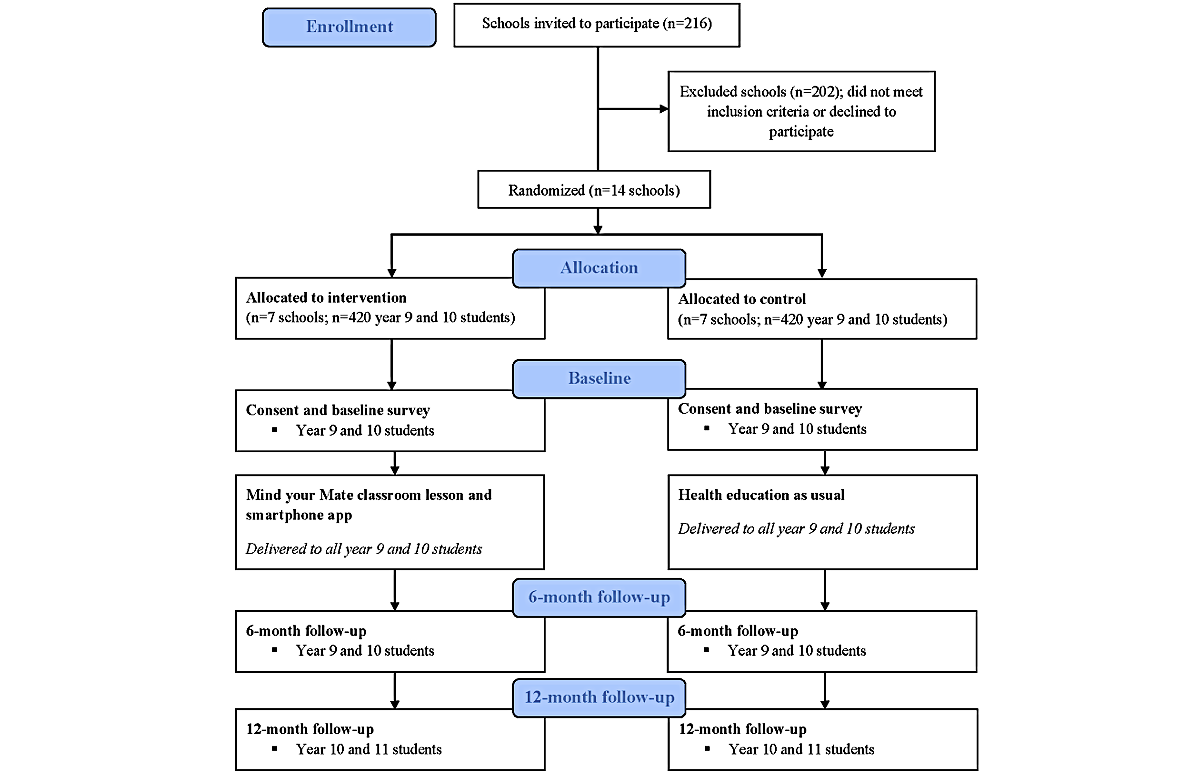
Anticipated recruitment, randomization, and assessment of participants based on the CONSORT (Consolidated Standards of Reporting Trials) guidelines.

To determine the effectiveness of the Mind your Mate intervention, a cluster RCT will be conducted among year 9 or year 10 students (aged approximately 14 years) at 14 independent and public secondary schools in NSW from 2020 to 2021. Cluster randomization will be used to avoid contamination of the controls by the intervention group through student communication. Schools will be randomly allocated to either the Mind your Mate intervention condition or the active control condition (health education as usual). Figure 1 summarizes the anticipated recruitment, randomization, and assessment of the participants.

### Sample Size Calculations

This trial is powered to detect intervention by time interactions in longitudinal cluster RCTs. To allow for comparisons between the two conditions, 6 schools (with at least 60 students) in each of the 2 intervention groups were required, giving a total of 12 schools (with at least 720 students). This would achieve 80% power to detect a standardized between-group mean difference of 0.2 in outcomes at the end of the trial with three measurement occasions (*P*=.05). An effect size of 0.2, which is comparable with previous trials of anxiety, depression, and substance use prevention programs [20,21,31-34], would have substantial benefits at the population level according to economic modeling [35]. It is anticipated that the majority of students in the year group of participating schools will take part in the study, based on participation rates found in previous school-based trials conducted by the research team [36-39]. To account for school dropouts during the trial, which are expected to be approximately 10% based on similar previous school-based trials [40], this study will recruit 14 schools (with at least 840 students).

### Procedure

#### Inclusion and Exclusion Criteria

Eligible participants will be all year 9 or year 10 students (aged approximately 14-15 years) attending participating schools that own a smartphone. Providing smartphones or laptops to study participants with technological barriers was not possible because of time and budget constraints. Mobile and internet literacy will be assumed. Students will be required to provide active informed consent, and only students with active parental consent will be eligible to participate.

#### Recruitment of Schools

A random selection of independent and public schools across the NSW Greater Sydney Area (Greater Capital City Statistical Area, 1 Greater Sydney) [41] will be approached through publicly available contact details. The study will be advertised through the researchers’ networks and the Matilda Center’s social media sites. Schools that took part in focus groups for feedback on mobile app development, as part of the Mind your Mate Development Study (University of Sydney Ethics Approval 2019/723) will also be approached. School principals will be sent an invitation letter via email outlining the aims of the study and seeking permission to implement the study in their school. Schools will then be followed up through phone calls by the research team.

Schools principals who consent to their school taking part will be asked to identify an appropriate staff member and inform the research team on how to liaise with this staff member with regard to the trial.

#### Randomization

Following the school principal’s approval, randomization and stratification according to gender will be conducted by a biostatistician external to the research team using the blockrand function in R (R Core Team). The school gender mix for stratification will be coeducational, predominately male, or predominately female, and the threshold for predominately male or predominately female will be at >60%. Half of the schools (n=7) will be randomly allocated to the Mind your Mate intervention condition and half (n=7) to an active control group (health education as usual). Similar to school-based interventions of this kind, students and teachers will not be blinded to intervention allocation.

#### Informed Consent

Participating schools will be asked to send information and active (opt-in) consent forms to parents or guardians of year 9 or year 10 students via hard copy or electronically. Students who receive parental consent will be provided with a web-based participant information statement and consent form and will be required to actively consent before beginning the study. Students who do not consent or who do not receive parental consent to participate in the research trial will still be offered access to the content of the program; however, they will not be prompted to complete assessment surveys, and no data will be collected from those individuals.

### Intervention

#### Overview

The Mind your Mate intervention was collaboratively designed by young people and experts in mental health and substance use prevention. Focus groups were conducted with year 10 students (n=23) from 2 independent secondary schools in Sydney to inform them of the critical components of the development of the mobile app and design. The Matilda Centre’s Youth Advisory Board, a group of young adults who provide feedback on youth-focused projects, guided the initial development and design of the app. Furthermore, each educational module within the app was reviewed by 2 psychologists with expertise on mental health and substance use. The wide range of input from a variety of ages and backgrounds was intended to ensure that the intervention is inclusive and tailored to the needs of adolescents.

Mind your Mate is an mHealth program aimed at upskilling and empowering adolescents to better support their peers about issues on mental health, alcohol, and other drug use. The intervention will include 1 introductory classroom lesson, led by a teacher but delivered on the web, and a downloadable mobile app. It is anticipated that the app will be made available to schools free of charge following the intervention.

#### Classroom Lesson

The Mind your Mate classroom lesson will be web-based through the study website by teachers of the participating schools. Teachers will be provided with an implementation guide containing instructions for accessing the survey, classroom lessons, and an outline of the content links to the NSW Personal Development, Health, and Physical Education Syllabus.

**Figure 2:**
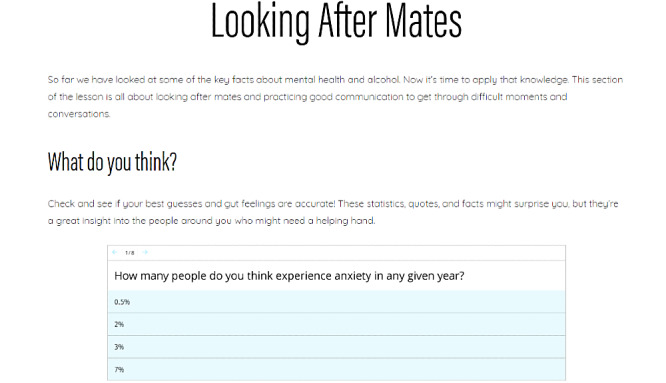
Screenshot of the Mind your Mate classroom lesson.

The classroom lesson will take approximately 40 minutes to complete and will be conducted after the students have completed the baseline survey. The lesson introduces common mental health and substance use problems encountered by young people and discusses the concept of supporting peers through animated examples, quizzes, and interactive activities (Figure 2 for example screenshots of these components). The lesson concludes with a video tour of the app’s features and instructions for students to download the app onto their mobile device. Students were instructed to use the mobile app as needed over the following 12-month period.

#### Mobile App

**Figure 3:**
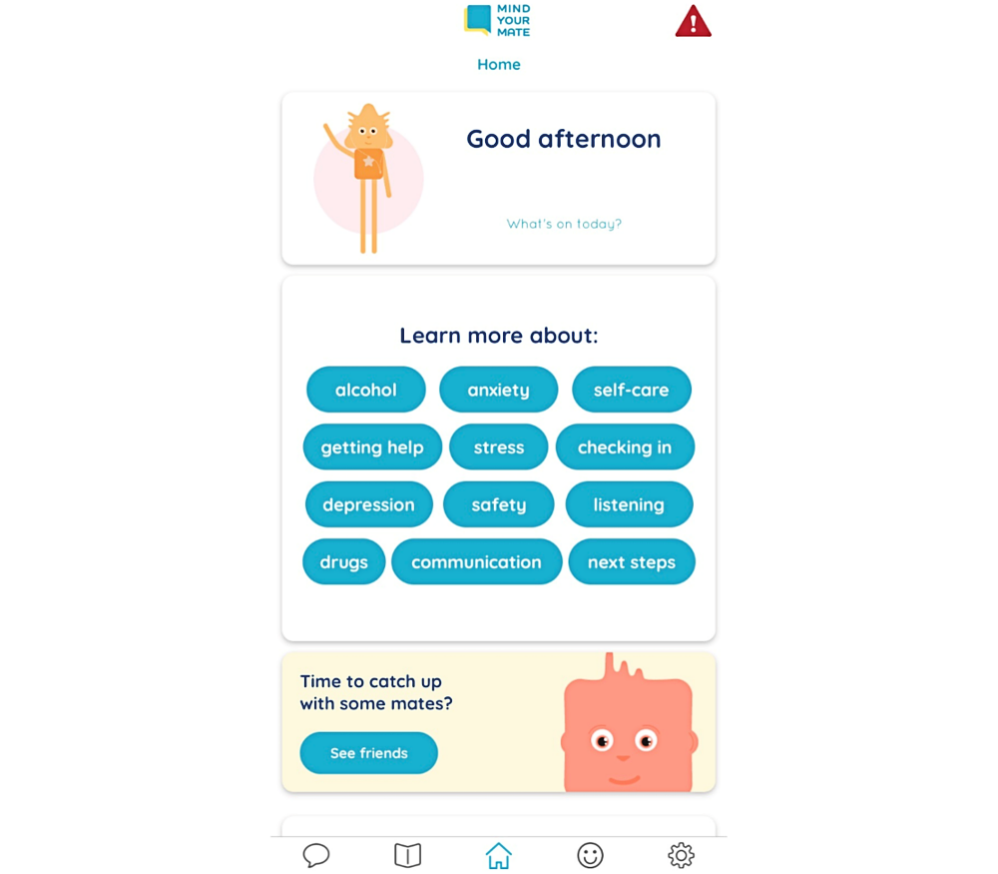
Screenshot of the Mind your Mate mobile app.

The Mind your Mate app aims to facilitate discussions among peers and provide key mental health literacy information about anxiety, depression, and substance use (Figure 3). It provides communication tools, educational modules, and suggested activities to engage in conversations and provide support for peers, including the ability to schedule conversations, follow-up, and example messages to send to peers. Several features were included to maintain engagement with the app based on feedback from the focus groups. These included emphasis on visual content such as infographics and videos rather than text, user experience customization through avatar selection and color scheme, gamification of content, and interactive features such as mood tracking and customized self-care activities. Automated pop-up reminder notifications were programmed to maintain their engagement. The reminders appear on the user’s phone home screen and are related to scheduled conversations with friends and the completion of the education modules. Digital metrics will be collected to measure engagement with the app, including uptake, frequency of usage, time spent in the app, and the features and modules that were accessed.

An overview of the Mind your Mate app content is provided in Table 1. The app is self-paced and only accessible to the participants in the intervention group in this study. Within the app, it is emphasized that students are not responsible for their friends or their friends’ choices. Students are encouraged to contact a responsible adult or health professional if they are concerned about their friends. Crisis helpline numbers are easily accessible on the home screen and under support options.

**Table 1:** Overview of the educational module content of the Mind your Mate smartphone app.

Module name	Key content covered	Related features
Mental health	Anxiety and depression literacy	Animated videos featuring personal stories Symptom diagrams Myth buster
Stressful times	Management of well-being References to COVID-19 pandemic and bushfires	Links to trusted external resources for self-help and emergencies
Alcohol and drugs	Substance use literacy, normative use, and standard drinks	Animated videos Symptom diagram Quiz question External link to standard drinks calculator
Listen up	Active listening skills Tips and tools to start conversations with friends	Scheduling conversations that link directly into calendar Example text to send to friends
Keeping friends safe	Harm minimization for substance use and supporting disclosure to parents or professionals	Recovery position diagram Video challenging fears of responsibility for a mate Harm minimization and emergency response video
Tricky conversations	Active questioning, motivational interviewing, and communication skills	Conversation preparation checklist and template Template messages to send to friends when reaching out Emotion dictionary
Checking in	Following up with friends and conversation tips	Scheduling follow-up conversations with friends directly into calendar Support links Profiles for friends to reference upcoming and past conversations
What next?	Challenges notion of having sole responsibility for another’s well-being and supporting disclosure to parents or professionals	Template to review conversation with friend Interactive checklist Textbox for reflection
Support options	Provision of formal and informal support options such as school counsellor, parent, free web-based resources, and general practitioners	Template messages to send to friends when reaching out Links to trusted external educational sites, resources, and support
Looking after yourself	Self-care Emotion regulation skills	Personalization of self-care activities list Ability to add custom activity Mood tracker

#### Active Control Group

Control schools will be asked to continue to implement their usual health and physical education classes for the 12-month trial period. The year 9 and year 10 Health and Physical Education curriculum mandates that content on alcohol and other drug use and well-being be implemented. As such, all control schools will implement curriculum-based health education during the trial. This group will serve as the *active control*. Although no app component will be offered to control schools during the trial, complimentary use of the intervention at the end of the study, if effective, will be available.

#### Assessment Occasions

All students (intervention and control conditions) will be asked to complete a web-based self-report survey in a supervised classroom setting at baseline and 6 and 12 months after baseline. Students completing web-based surveys will be assigned a unique participant identifier generated in REDCap (Research Electronic Data Capture; Vanderbilt University), a secure web-based data collection system. This identifier will be used to email students with personalized survey links that they will use to complete each survey. This unique code links student data over time while maintaining confidentiality. Absent students will be contacted by the research team (using details provided upon registration) and invited to complete the survey at a later date. Students will be made aware that all the contact information they provide will be kept strictly confidential and secure, separate from their survey responses. After each survey, students will be incentivized with entry into a draw for 1 of 5 Aus $50 (US $38) Prezzee gift vouchers (5 vouchers per assessment occasion).

### Measures

#### Overview

Demographic data, including age, sex, gender, home postcode, and country of birth, will be obtained to determine the baseline equivalence of the groups. To assess academic performance, students will nominate the range that their grades usually fall into (ie, <49%, 50%-59%, 60%-69%, 70%-79%, 80%-89%, and 90%-100%). Truancy will be measured by students reporting the number of days that they had off from school in the previous year without their parents’ permission.

#### Primary Measures

##### Knowledge

Mental health and substance use knowledge specific to content within the mobile app will be assessed using a questionnaire developed by the research team (Multimedia Appendix 1). General mental health literacy will be assessed using questions adapted from the Mental Health Literacy Questionnaire [42].

##### Alcohol and Other Substance Use

Drinking behavior in the past 6 months will be assessed by using questions adapted from the School Health and Alcohol Harm Reduction Project Patterns of Alcohol index [43] and reflect those used in previous trials the team has led [44]. Students will be asked to report the frequency and quantity of alcohol consumption in standard drinks, proportion of friends who drink, and intentions to try alcohol. A pictorial standard drink chart will be supplied within the survey to assist students in identifying what constitutes a standard drink in common portions and the types of alcohol [45].

The reasons for not drinking alcohol will be assessed using the Motives for Abstaining from Alcohol Questionnaire [46]. Students will be asked to rate how important each statement is to them personally, with response options ranging from *not at all important* to *extremely important*.

Other substance use will be assessed using questions adapted from the Australian Institute of Health and Welfare: National Drug Strategy Household Survey [47]. Students will be asked to indicate whether they have ever tried tobacco, vaping, cannabis, ecstasy, cocaine, or methamphetamines; the nonmedical use of prescription drugs; or a combination of drugs at the same time. Students will further be asked how likely it is that they will try any of the listed drugs in the future and the proportion of friends and acquaintances who use drugs.

##### Mental Health Measures

Psychological distress in the past month will be assessed using the Kessler 6 scale [48]. Depression and anxiety symptoms during the past 2 weeks will be measured using the Patient Health Questionnaire 8 [49] and the Generalized Anxiety Disorder 7-item scale [50], respectively.

##### Help-Seeking Measures

The General Help-Seeking Questionnaire [51] will be used to assess future help-seeking behavioral intentions from various sources. A list of potential sources of help will be shown. Students will first be asked to indicate how likely it is that they would seek help from each of these sources for personal or emotional problems, then how likely it is that they would seek help from each of these sources if they were experiencing suicidal thoughts. Responses will be given using a 7-point scale ranging from *extremely unlikely* to *extremely likely*.

Recent help-seeking behavior in the past 2 weeks will be measured using the Actual Help-Seeking Questionnaire [52] and questions adapted from the Mission Australia Report, 2018 [53]. Students will be asked to indicate whether they have sought help from a list of sources in the past 2 weeks. If they select *yes*, they will be asked to briefly describe the type of problem that they sought help for.

#### Secondary Measures

##### Positive Well-being

The impact of the intervention on positive well-being will be assessed using measures adapted from the Warwick-Edinburgh mental well-being scale [54]. Students will be asked to read three statements, such as “I have been feeling good about myself,” and tick the response that best reflects their experience of that statement in the past 2 weeks. Response options range from *none of the time* to *all of the time*.

##### Quality of Life

The Pediatric Quality of Life questionnaire [55] will be used to measure participants’ self-reported quality of life. Students will be asked to complete the questions from the perspective of their own health that day, such as “I don’t feel worried today.”

##### Impact of COVID-19 Pandemic

The impact of the COVID-19 pandemic on physical and mental health will be assessed using measures adapted from the Australian National COVID-19 Mental Health, Behaviour and Risk Communication Survey [56].

### Fidelity and Implementation Log

Students in the intervention group will be asked to provide feedback on their experience and the use of the mobile app, as well as suggestions they may have for improvement, at the 6- and 12-month follow-up occasions. In addition, one teacher at each participating school will be asked at the 12-month follow-up to complete a logbook documenting any additional mental health or alcohol and drug education that was implemented over the course of the study. The logbook will be used to control for the effects of additional well-being programs. Digital metrics of mobile app engagement will also be collected.

### Statistical Analysis

Analyses will use an intention-to-treat approach, including all available measurements for all students, in the groups in which they were randomized. Baseline equivalence and attrition between groups will be examined using single-level analyses, one-way analyses of variance to examine normally distributed data, chi-square analyses to examine binominal data, and Mann-Whitney *U* tests to examine nonnormally distributed data. The baseline data of the primary outcomes will be used as covariates to ensure that any differences between groups at baseline are accounted for. To examine intervention by time interaction–effects, mixed-effects regression will be used because of the multi-level nature of the data (students nested within schools). Hypothesized intervention effects will primarily be examined using mixed-effects linear regression analyses for normally distributed data and mixed-effects logistic regression analyses for categorical data. Bonferroni adjustments will be made for multiple comparisons where appropriate. Multiple imputation and regression weighting strategies will be used to examine the impact of attrition. Sensitivity analyses will be used to assess the effect of attrition on inferences drawn from the target parameters in the statistical analyses**.**

## Results

This study was funded by an Australian Rotary Health Bruce Edwards Postdoctoral Research Fellowship from 2019 to 2022. Some of the development costs for the Mind your Mate intervention came from a seed funding grant from the Brain and Mind Centre, The University of Sydney. The enrollment of schools began in July 2020, with 12 of 14 schools enrolled at the time of submission. Baseline assessments are currently underway, and the first results are expected to be submitted for publication in 2022.

## Discussion

### Principal Findings

This protocol outlines the design of a cluster RCT to evaluate the effectiveness of an mHealth peer intervention for preventing mental health symptoms and alcohol and other drug use and promote help seeking and knowledge among adolescents. To our knowledge, the proposed intervention is the first peer-prevention program delivered via a mobile app facilitated through the school system. If effective, it has the potential for widespread implementation at a relatively low cost, as it uses a delivery method that is both acceptable and easily accessible to adolescents.

### Strengths and Limitations

The key strengths of the proposed effectiveness study are the intervention’s focus on fostering peer support among adolescents and the use of digital technology. The principle of peer support embedded within the intervention aims to strengthen and foster adolescents’ social connections both within their peer groups and with trusted adults. Social connection is known to be a key protective factor for mental health problems [57,58]. Furthermore, help seeking for mental health, drug, and alcohol concerns has long been plagued by surrounding stigma and a lack of knowledge of relevant support services; thus, most young people do not seek support when it is needed [24,59]. The provision of easily accessible information in the Mind your Mate smartphone app that destigmatizes common mental health challenges and provides support options might help overcome some of these barriers and encourage early help seeking in adolescents. Indeed, programs aimed at reducing mental health stigma are associated with increased confidence among young people in supporting peers [27]. Future research may more closely examine the interaction between stigma and peer-based processes.

Web-based delivery and the use of a mobile app means that the intervention is easily accessible and uses a delivery platform with which adolescents are familiar and comfortable. It also provides the potential to deliver the intervention widely at a relatively low cost, as no teacher training is required. The app itself is self-paced and can be used by students in their own time, with only a short time requirement anticipated for teachers. Furthermore, the web-based mode of delivery of the intervention ensures the fidelity of high-quality information and facilitates ease of access.

Another benefit of the program is its links to existing school health and physical education curricula, meaning schools could act as key delivery partners to roll out the intervention if found to be effective. Finally, the intervention was collaboratively designed with young people, meaning it is more likely to be acceptable and engaging for young people, an important outcome not only within the context of the current trial but also for future efforts to take the program to scale.

One limitation of the study is the reliance on participant self-report to collect data for the primary outcomes of this trial. Although these were chosen due to ethical and feasibility considerations and these are standard practices in school-based trials of mental health and substance use prevention [20,60], it is possible that measures of mental health and/or substance use could be subject to underreporting or overreporting. To mitigate these effects, students are reminded of the confidentiality of their responses at each survey occasion and encouraged to be as honest as possible, thereby reducing the risk of bias or concealment. Owing to time and budget constraints, the study was unable to provide smartphones to students who did not own them. Future research may adapt the intervention to be suitable for a range of groups, including adolescents living in rural and regional areas and adolescents experiencing technology barriers such as lack of access to smartphones and computers. Furthermore, although there is some evidence that digital tools may be more effective for certain users when human support is included compared with standalone tools [61], this was beyond the scope of this study because of time and budget constraints. Future research may explore the addition of human support or check-ins for peer-led mHealth programs and their impact on outcomes and user engagement. Finally, given the age of the students in the study (14-15 years old), we may be limited in our ability to assess change in substance use over the period of the trial, as the proportion of students using substances at this age may be small and typically tends to increase in later adolescence. Nonetheless, the Mind your Mate intervention was developed as a prevention approach because of the importance of early intervention, before thoughts and behaviors have become entrenched.

### Conclusions

The development of a novel mHealth tool for young people to support their peers and improve their own mental health represents an innovative applied approach to the prevention of mental disorders in a real-world setting. If effective, the intervention is low cost and will link to the existing high school syllabus, with the potential for widespread implementation and significant impact.

## Appendix

Multimedia Appendix 1 App-specific knowledge questionnaire.

## Abbreviations

CONSORT: Consolidated Standards of Reporting Trials
mHealth: mobile health
NSW: New South Wales
RCT: randomized controlled trial
REDCap: Research Electronic Data Capture
