# Supplementary material for: A Mobile Peer Intervention for Preventing Mental Health and Substance Use Problems in Adolescents: Protocol for a Randomized Controlled Trial (The Mind Your Mate Study)
Source: JMIR Res Protoc. 2021 Jul 30;10(7):e26796. doi: 10.2196/26796 (PMC8367112; doi:10.2196/26796)
Supplement: Multimedia Appendix 1 [file resprot_v10i7e26796_app1.docx]

**Multimedia Appendix 1.** App-specific knowledge questionnaire.

**Please indicate your answer to each question below by selecting True, False, or ‘Don’t Know’:**

| 1. The Australian guidelines say that for people **under the age of 18**, the safest option is to **not drink alcohol at all**. | **True** | False | DK |
| --- | --- | --- | --- |
| 1. Adults can have up to **4 standard drinks** on one day without greatly increasing their risk of alcohol-related injury or disease. | **True** | False | DK |
| 1. The brain is still developing **until you are 25 years old**. | **True** | False | DK |
| 1. Being an **active listener** means listening briefly to a friend, then offering solutions to their problems. | True | **False** | DK |
| 1. Over **half** of young people aged 12-17 years old have **had a full alcoholic drink**. | True | **False** | DK |
| 1. A pounding heart, feeling sick and sweating can all be symptoms of **anxiety**. | **True** | False | DK |
| 1. Everyone feels anxious or low from time to time. | **True** | False | DK |
| 1. Having **more energy** and wanting to **see your friends all the time** is a symptom of depression. | True | **False** | DK |
| 1. When people feel **depressed,** they might commonly think that “there is no point to anything” and feel worthless. | **True** | False | DK |
| 1. When listening to a friend who is going through a hard time it is a good idea to **dismiss their feelings** and say something like, “you just need to stop worrying so much”. | **True** | False | DK |
| 1. If your friend has drunk too much alcohol or taken a drug you should leave them on their own. | True | **False** | DK |
| 1. You should act as a personal counsellor to your friend if they have no one else to talk to. | True | **False** | DK |
